# Activation of Thiazide-Sensitive Co-Transport by Angiotensin II in the *cyp1a1-Ren2* Hypertensive Rat

**DOI:** 10.1371/journal.pone.0036311

**Published:** 2012-04-27

**Authors:** Ali Ashek, Robert I. Menzies, Linda J. Mullins, Christopher O. C. Bellamy, Anthony J. Harmar, Christopher J. Kenyon, Peter W. Flatman, John J. Mullins, Matthew A. Bailey

**Affiliations:** 1 University/British Heart Foundation Centre for Cardiovascular Science, The University of Edinburgh, Edinburgh, United Kingdom; 2 Centre for Integrative Physiology, The University of Edinburgh, Edinburgh, United Kingdom; 3 Department of Pathology, Edinburgh New Royal Infirmary, Edinburgh, United Kingdom; Max-Delbrück Center for Molecular Medicine (MDC), Germany

## Abstract

Transgenic rats with inducible expression of the mouse *Ren2* gene were used to elucidate mechanisms leading to the development of hypertension and renal injury. *Ren2* transgene activation was induced by administration of a naturally occurring aryl hydrocarbon, indole-3-carbinol (100 mg/kg/day by gastric gavage). Blood pressure and renal parameters were recorded in both conscious and anesthetized (butabarbital sodium; 120 mg/kg IP) rats at selected time-points during the development of hypertension. Hypertension was evident by the second day of treatment, being preceded by reduced renal sodium excretion due to activation of the thiazide-sensitive sodium-chloride co-transporter. Renal injury was evident after the first day of transgene induction, being initially limited to the pre-glomerular vasculature. Mircoalbuminuria and tubuloinsterstitial injury developed once hypertension was established. Chronic treatment with either hydrochlorothiazide or an AT1 receptor antagonist normalized sodium reabsorption, significantly blunted hypertension and prevented renal injury. Urinary aldosterone excretion was increased ∼20 fold, but chronic mineralocorticoid receptor antagonism with spironolactone neither restored natriuretic capacity nor prevented hypertension. Spironolactone nevertheless ameliorated vascular damage and prevented albuminuria. This study finds activation of sodium-chloride co-transport to be a key mechanism in angiotensin II-dependent hypertension. Furthermore, renal vascular injury in this setting reflects both barotrauma and pressure-independent pathways associated with direct detrimental effects of angiotensin II and aldosterone.

## Introduction

Hypertension is a major world health problem associated with a substantial cost, in terms of patient mortality/morbidity and economic burden. Tremendous strides have been made in this field and there are several treatments for reducing blood pressure in most patients. Nevertheless, basic research to establish causal pathways may lead to more effective management of existing hypertension or importantly provide a foundation for life-style advice in the pre-hypertensive phase. This endeavor is facilitated by the use of appropriate animal models. One such model is the *cyp1a1*-*Ren2* transgenic rat (TGR) in which hypertension can be reversibly induced, without surgical intervention, by dietary administration of the non-toxic, naturally occurring (for example in brassicas) aryl hydrocarbon, indole-3-carbinol (I3C) [1]. An advantage of this model is that changing the dose of I3C administered can alter the severity of hypertension. This allows, for instance, the generation of a slowly-developing hypertension, analogous to chronic infusion of subpressor doses of angiotensin II [2], [3] or malignant hypertension (MH), characterized by rapidly accelerating blood pressure and injury to target organs [4].

In the *cyp1a1*-*Ren2* rat, a transgene has been integrated into the Y chromosome. This transgene places mouse *Ren2* cDNA expression under the control of an inducible cytochrome p450-1a1 promotor [1]. Expression of *Ren2*, primarily in the liver, leads to increased circulating renin levels, activation of the renin-angiotensin-aldosterone system and a rise in blood pressure. This rise in pressure is accompanied by a sustained elevation of circulating renin. Intrarenal synthesis of angiotensin II [5], [6], promoted by elevated renin and pro-renin receptor expression [7], contributes to the pathology of hypertension in the *cyp1a1*-*Ren2* rat, and the model is therefore complementary to approaches using chronic infusion of angiotensin II, in which renin activity is suppressed.

The current study was designed to resolve key mechanisms leading to hypertension in the *cyp1a1-Ren2* TGR and focuses on sodium transport in the distal convoluted tubule. Several lines of evidence led us to hypothesize that hypertension in the TGR model originates in the kidney and reflects an impaired ability to excrete sodium. First, dietary sodium loading exacerbates hypertension [5]. Second, renal blood flow is attenuated [4], pressure natriuresis is impaired [8] and tubuloglomerular feedback is activated [9], whilst third, the systemic hormonal profile of elevated angiotensin II and aldosterone would reduce natriuretic capacity by effects on the tubule epithelium [10], [11], [12], [13] and medullary *vasa recta* [14]. Our hypothesis is further supported by the observation that manoeuvers improving renal blood flow and/or sodium excretion blunt the hypertensive response to *Ren2* activation [15], [16].

We find that induction of hypertension in the *cyp1a1-Ren2* TGR causes sustained activation of the thiazide-sensitive co-transporter by angiotensin II in the distal tubule. Renal injury is predominantly vascular, precedes the development of hypertension and may involve mineralocorticoid-dependent pathways.

## Results

**Figure 1:**
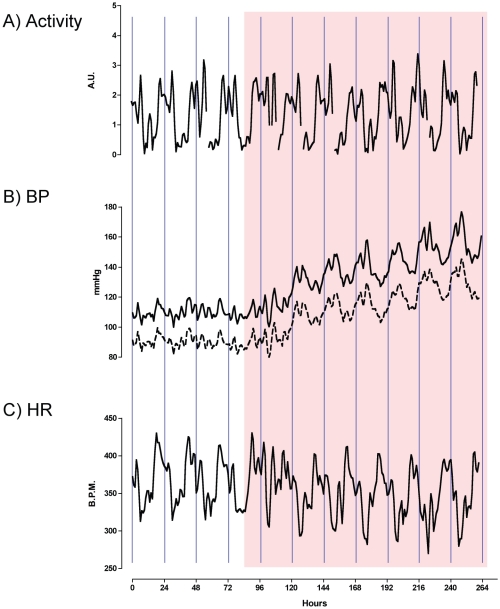
Telemetry data from *Cyp1a1-Ren2* transgenic rats. Recordings were made in rats (n = 5) over a baseline period and following 7 consecutive days of indole-3-carbinol administration (I3C; Shaded area). A) locomotor activity; B) Blood pressure (BP), with systolic as a solid line and diastolic as a dashed line and C) HR. Data are hourly means, smoothed with a 5-point rolling average and shown without error bars for clarity. The vertical lines represent the midnight time point. I3C was administered at 10am.

Systolic blood pressure and diastolic blood pressure increased significantly during the 24 hours following the second dose of I3C (Figure 1). Over the experimental time-course, systolic increased proportionally more than diastolic blood pressure and pulse pressure was therefore increased. Heart rate fell significantly during the period of transgene induction, consistent with an intact baroreceptor reflex. The day-night cycle of locomotor activity was unaffected by RAAS activation. The statistically significant periodicity of blood pressures, heart rate and activity was approximately circadian during the baseline period. Analysis was performed on de-trended data from the hypertensive period and persistency of circadian rhythmicity was observed.

### Activation of the cyp1a1-Ren2 transgene promotes anti-natriuresis

**Figure 2:**
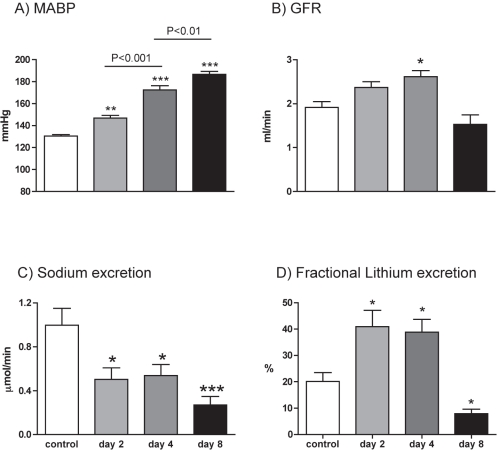
Renal function in anaethetized rats. A) mean arterial blood pressure (MABP); B) glomerular filtration rate (GFR); C) urinary sodium excretion and D) the fractional excretion of lithium. Measurements were made in *cyp1a1-Ren2* transgenic rats, on either day 2 (n = 9), 4 (n = 9) or 8 (n = 9) in the induction regimen. Non-induced rats (n = 8) served as control. Data are mean ± SE. Statistical comparisons were made using ANOVA with Bonferroni post-test. ***P<0.001, **P<0.01, *P<0.05 versus the control group.

Under anaesthesia, we observed a progressive increase in mean arterial blood pressure (Figure 2A, ANOVA P<0.001) with repeated doses of I3C, with a small but significant rise being observed on day 2 of *Ren2* induction. Glomerular filtration rate rose significantly, at least until day 4 (Figure 2B; ANOVA P<0.01) but then fell back to control levels. Effective renal plasma flow (Table 1) was stable. In consequence, filtration fraction was elevated only on day 4 and renal vascular resistance only on day 8(Table 1). Transgene induction reduced natriuretic capacity (Figure 2C; ANOVA P<0.001), with sodium excretion falling to ∼50% of control values by day 2. There was a significant linear trend toward hypokalaemia (P<0.05), despite which fractional potassium excretion remained robust (Table 1).

**Table 1:** Renal data and plasma electrolytes in *cyp1a1*-*Ren2* transgenic rats before and after the induction of hypertension.

	Control	Day 2	Day 4	Day 8	ANOVA
n	8	9	9	8	
Body weight (g)	324±14	318±16	343±13	305±13	NS
FE_Na_ (%)	0.39±0.06	0.16±0.03^**^	0.15±0.03^**^	0.09±0.02^**^	<0.001
FE_K_ (%)	38.0±4.5	30.3±2.9	31.7±2.2	30.0±9.4	NS
V (µl/min)	9.8±0.7	13.6±3.5	13.2±1.0	9.3±2.0	NS
P_Na_ (mmol/l)	135.9±0.4	134.3±0.7	134.0±0.6	132.6.3±1.7	= 0.059
P_K_ (mmol/l)	4.23±0.06	4.10±0.12	4.03±0.11	3.87±0.09	NS
Hct (%)	46.5±1.0	48.3±0.5	48.7±1.0	52.0±1.1^**^	<0.01
eRPF (ml/min)	6.7 ± 0.6	8.3±0.8	7.2±0.4	7.6±1.0	NS
RBF (ml/min)	12.7±0.7	16.0±1.3	14.0±0.7	16.1±2.1	NS
FF (%)	28.4±1.6	29.8±2.2	37.0±2.0^**^	20.0±1.5^*^	<0.01
RVR (mmHg.ml.min−1)	10.5±0.6	9.7±0.8	12.4±0.6	14.4±1.3^*^	<0.01

Data are mean± SE. Comparisons were made using one-way ANOVA (P value shown in column), with Bonferroni post-hoc test: * = P<0.05, ** = P<0.01. FE_Na_ and FE_K_ indicates the fractional excretion of sodium and potassium; V is urine flow rate; P_Na_ and P_K_ are the plasma concentration of sodium and potassium; Hct is hematocrit; eRPF and RBF are effective renal plasma and blood flow, respectively; FF is filtration fraction and RVR is renal vascular resistance.

Fractional sodium excretion fell (Table 1) with transgene induction, indicating a tubular origin for the antinatriuresis. We attempted to localize this effect by measuring the fractional excretion of lithium excretion (Figure 2D). This was initially elevated (ANOVA P<0.01), indicating diminution of proximal tubular reabsorption and localizing the antinatriuretic effect to more distal nephron segments.

**Figure 3:**
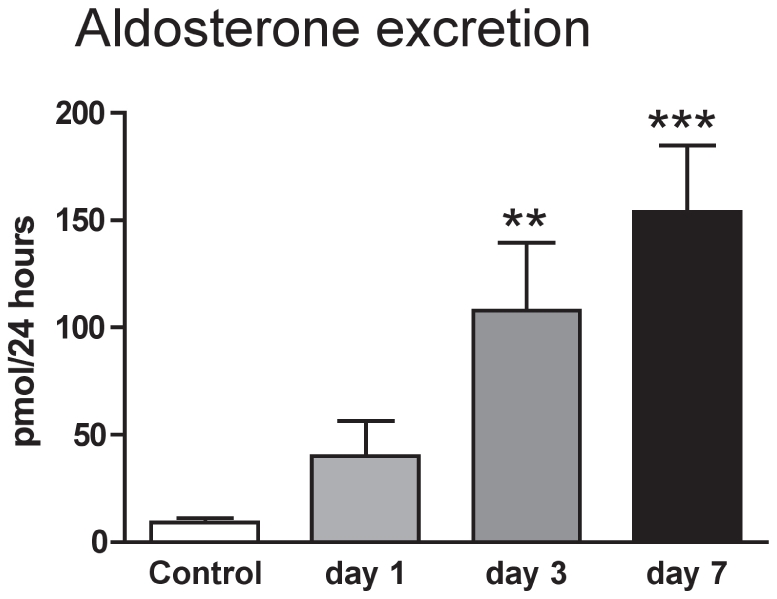
24-hour urinary excretion of aldosterone. Urine was collected from conscious *cyp1a1-Ren2* transgenic rats (n = 8) maintained in individual metabolism cages, over consecutive days of transgene induction. Data are mean ± SE. Statistical comparisons were made using ANOVA with Bonferroni post-test. ***P<0.001, **P<0.01 versus the control day.

**Figure 4:**
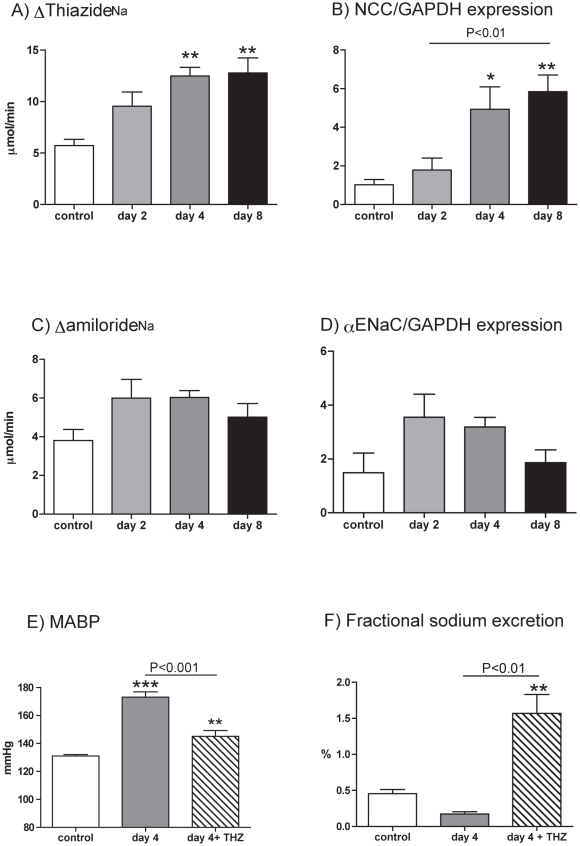
Sodium transport pathways in the aldosterone-sensitive distal nephron. A) hydrochlorthiazide-sensitive sodium reabsorption (Δthiazide_Na_); B) the expression of the thiazide-sensitive co-transporter protein (NCC) in whole kidney extracts, normalized to that of GAPDH; C) amiloride-sensitive sodium reabsorption (Δamiloride_Na_); D) the expression of the α subunit of the epithelial sodium channel (αENaC) normalized to that of GAPDH. Measurements were made in *cyp1a1-Ren2* transgenic rats, on either day 2 (n = 9), 4 (n = 9) or 8 (n = 9) in the induction regimen. Non-induced rats (n = 8) served as control. E) mean arterial blood pressure (MABP) and F) fractional sodium excretion. Measurements were made in *cyp1a1-Ren2* transgenic rats receiving either vehicle (grey bar) or hydrochrorothaizide (hatched bar) by minipump. Data are mean ± SE. Statistical comparisons were made using ANOVA with Bonferroni post-test. P<0.001, **P<0.01, *P<0.05 versus the control group.

In conscious rats, sodium intake (initially 2.38±0.11 mmol/24 h), declined over the induction period but this was not statistically significant until the final day (1.38±0.17 mmol/24 h; P<0.01). Body weight was found not to change significantly, with end-weight being 96.5±1.2% of start weight. 24 h urinary aldosterone excretion was increased ∼20 fold over the induction period (Figure 3 ANOVA P<0.001) and we therefore focused our experiments on the aldosterone-sensitive distal nephron (ASDN). Sodium-chloride co-transport (NCC, *Slc12a3*) and the epithelial sodium channel (ENaC, *Scnn1*) account for the majority of sodium transport in the ASDN and the contribution of each was assessed pharmacologically. At baseline, thiazide-sensitive transport was responsible for the reabsorption of ∼5% of the filtered sodium load. This increased progressively during *Ren2*-transgene induction (Figure 4A; ANOVA P<0.01), as did abundance of total NCC protein relative to GAPDH (Figure 4B; ANOVA P<0.001). A positive correlation (Pearson r = 0.60; P<0.01) was observed between NCC protein abundance and thiazide-sensitive sodium reabsorption. An amiloride-sensitive pathway reabsorbed ∼2% of the filtered sodium in non-induced rats. There was a slight initial increase in both amiloride-sensitive sodium reabsorption (Figure 4C) and in the abundance of αENaC relative to GAPDH (Figure 4D). Although neither reached statistical significance, both sets of data suggest a similar trend- a transient rise, with both back to baseline by day 8.

The role of NCC-mediated sodium reabsorption was assessed by chronically administering thiazide during transgene induction. The blood pressure increase was significantly attenuated (Figure 4E), but still remained significantly higher than control animals. The partial rescue of the hypertensive phenotype was associated with a large increase in fractional sodium excretion (Figure 4F). An acute bolus of hydrochlorothiazide produced no further natriuretic effect (data not shown), confirming that NCC blockade was complete at the chronic infusion level.

### Activation of the cyp1a1-Ren2 transgene causes microvascular injury

**Figure 5:**
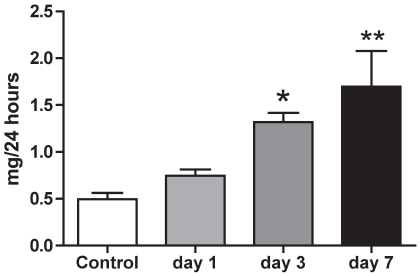
24-hour urinary excretion of albumin. Urine was collected from conscious *cyp1a1-Ren2* transgenic rats (n = 8) maintained in individual metabolism cages, over consecutive days of transgene induction. Data are mean ± SE. Statistical comparisons were made using ANOVA with Bonferroni post-test. **P<0.01, *P<0.05 versus the control day.

**Figure 6:**
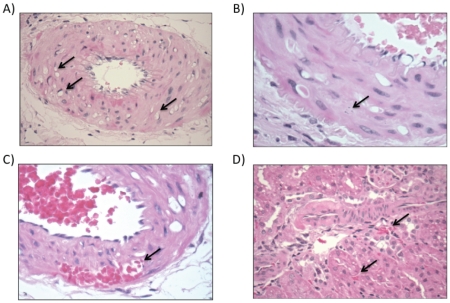
Temporal progression of microvascular injury in kidneys from *cyp1a1-Ren2* transgenic rats. (A) After 1 day of transgene activation myocycte vacuolation was observed in the media of the larger arteries (indicated by arrow). (B) After 3 days apoptotic nuclear fragments were observed (arrow) and (C) there were areas of hemorrhage into necrotic foci. (D) After 7 days, vasculopathy was more extensive still and there were foci of tubulointerstitial injury.

Modest albuminuria developed over the experimental time-course (Figure 5; ANOVA P<0.001) and kidneys were therefore examined for microvascular injury. An ordered categorical scoring of microvascular injury indicated a significant (X^2^ analysis; P = 0.028) contingency between the duration of transgene induction and microvascular injury. After 1 day of treatment, medial myocyte vacuolation was evident in arcuate and the larger interlobular arteries, indicative of vasospasm (Figure 6A). There was also rare focal medial myocyte cell death (Figure 6B). No such injury was observed in the control group. In rats studied at day 4, vascular damage was both more prevalent and severe, with foci of confluent medial myocyte death, apoptotic nuclear fragments and hemorrhage into the necrotic foci (Figure 6C). There was low-grade mononuclear cell infiltration into the perivascular adventitia. By day 8, the destructive vascular injury was more extensive still (Figure 6D), and fibroid necrosis was also evident in smaller interlobular arteries and afferent arterioles, consistent with our previous data [4]. Areas of tubuloinsterstitial injury were also observed, which were small and localized mainly in the cortex. There was no histological evidence of malignant phase hypertension, such as “onion-skinning” of the renal arterioles.

### Effect of spironolactone or losartan on blood pressure, renal function an microvascular injury

**Figure 7:**
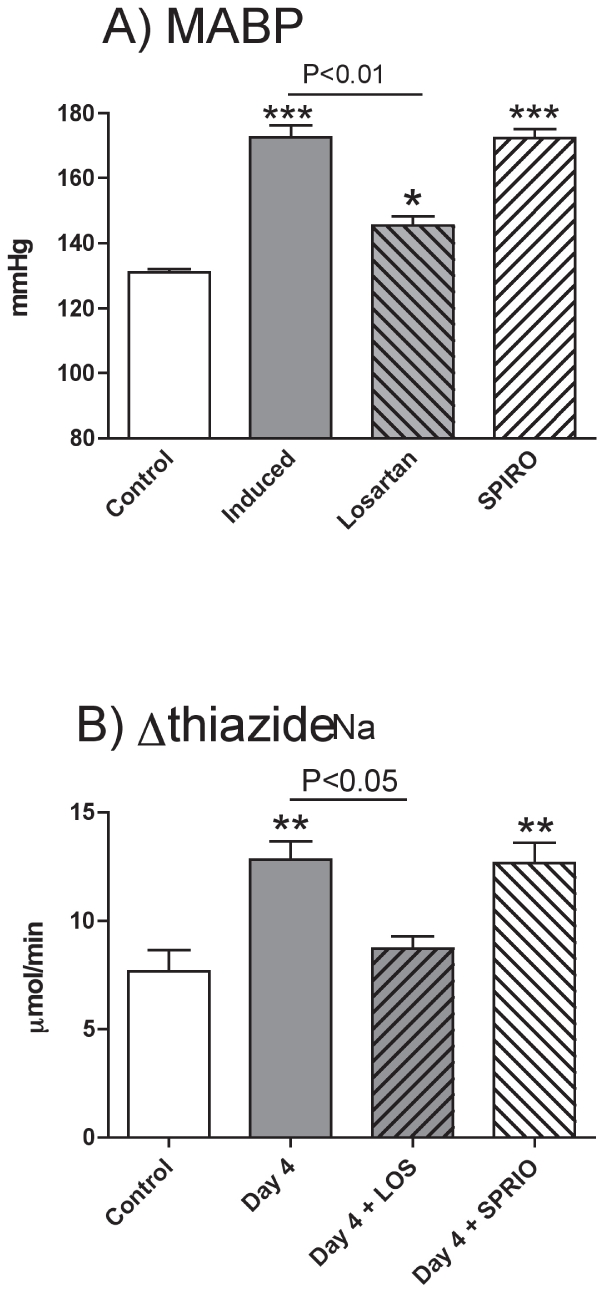
Contributions to hypertension of AT1 and mineralocorticoid receptors. A) Mean arterial blood pressure (MABP) and B) hydrochlorthiazide-sensitive sodium reabsorption (Δthiazide_Na_) in *cyp1a1-Ren2* transgenic rats. Measurements were made in cyp1a1-Ren2 transgenic rats, anaesthetized on day 4 of the experimental regimen. The first control group (n = 6; open bars) received vehicle, the second control group (n = 8; grey bars) received I3C to induce the Ren2 transgene. Experimental groups received I3C and either losartan (n = 6; grey hatched bars) or spironolactone (n = 6; open hatched bars). Data are means ± SE and statistical comparisons were made using ANOVA with Bonferroni post-test. ***P<0.001, **P<0.01, *P<0.05 versus the non-induced control group and other comparisons as stated.

Chronic administration of losartan blunted the hypertensive response to transgene induction (Figure 7A), increased fractional sodium excretion (*Ren2* induction alone = 0.15±0.03% versus co-administration of losartan = 0.40±0.04; P<0.01) and normalized thiazide-sensitive sodium reabsorption (Figure 7B). Kidneys from three of these rats were examined histologically and there was no evidence of hypertensive vascular injury. Spironolactone had no antihypertensive effect (Figure 7A) and nor did it have any effect on thiazide-sensitive sodium reabsorption (Figure 7B). Mineralocorticoid receptor blockade did, however, prevent the development of albuminuria during transgene induction (albumin excretion in mg/24 h) *Ren2* induction alone = 1.16±0.12; *Ren2* induction with co-administration of spironolactone = 0.18±0.07; P<0.01). Kidneys from five spironolactone treated rats were examined histologically: in two cases there was no evidence of vascular injury; in the other three, variable fibroid necrosis was observed.

## Discussion

In this study we investigated the early renal adaptation to RAAS activation in the *cyp1a1-Ren2* TGR. We show that the early phase of hypertension, characterized in this model by circulating angiotensin II in the high physiological range [5], is associated with impaired natriuresis due to activation of the thiazide-sensitive sodium-chloride cotransporter, with little contribution from amiloride-sensitive sodium transport.

### Hypertension, sodium reabsorption and thiazide-sensitive co-transport

The synergistic effect of angiotensin II and sodium intake on blood pressure has long been known but underlying mechanisms are not fully understood. Angiotensin II promotes sodium retention and volume expansion, particularly if sodium intake is high [17], [18], [19]. However, a strong correlation between sodium balance and blood pressure is not a consistent feature [19], [20], perhaps because increased renal arterial pressure can stimulate natriuresis [21]. Sustained hypertension may also reflect salt-induced sensitization of the sympathetic nervous system [22] and vascular smooth muscle [23] to angiotensin II.

Previous studies in *cyp1a1-Ren2* TGR have demonstrated that hypertension is associated with enhanced sodium reabsorption [6] and the rise in blood pressure is aggravated by dietary sodium-loading [5]. In the current study, sodium intake was lower by ∼10% over days 1–4 of transgene induction and reduced by 40% by the final day. This might contribute to a reduced sodium excretion, particularly in rats studied after 8 days of hypertension. Nevertheless, body weight was stable and in the cross-sectional studies plasma sodium was not changed. In this setting we observed an impaired capacity to excrete an intravenous saline load. In the proximal tubule, angiotensin II exerts a biphasic effect on sodium transport, becoming inhibitory at high concentrations [24], [25]. In our study, the increase in fractional lithium excretion observed at days 2 and 4 indicates a *reduction* in proximal tubule sodium reabsorption [26] and localizes the antinatriuresis to more distal nephron segments. Since mice infused chronically with angiotensin II display increased sodium reabsorption in the ASDN [27], we probed the function of this region with inhibitors of the major pathways for sodium transport, ENaC and NCC. Both angiotensin II and aldosterone can stimulate ENaC activity [13], [28] but we found the amiloride-sensitive pathway small and not greatly influenced by transgene induction. There was perhaps a trend towards an increase in both amiloride-sensitive transport and αENaC protein abundance but this normalized by day 8. Our protocol ultimately causes malignant hypertension after ∼14 days [4]. In contrast, when induction is aimed at producing a non-malignant stable hypertension, chronic amiloride treatment causes a transiently negative sodium balance and abolishes hypertension in *cyp1a1-Ren2* TGR [3], even though ENaC mRNA levels do not change. It is plausible that that modest increases in angiotensin II activate existing pools of ENaC and the resulting increase in blood pressure is amiloride-sensitive. However, when angiotensin II levels are increased further, a sustained increase in NCC activity is observed. The increased sodium reabsorption in the DCT would reduce sodium delivery to the collecting duct and thereby limit amiloride-sensitive sodium transport. Under such circumstances, hypertension would be expected to be mainly thiazide-sensitive (Figure 4E). The thiazide-resistant component in the present study was small (Figure 4E) and may be due to the transiently increased ENaC activity, uncovered when NCC is inhibited.

Thiazides are considered to be selective inhibitors of transport by NCC, but recently it has been shown that they also inhibit sodium reabsorption through *Slc4A8* in the collecting duct [29]. However, the strong positive correlation between NCC abundance and thiazide-sensitive sodium reabsorption suggests that transport through *Slc4A8* plays only a minor role in our studies.

In order to be physiologically active, NCC must be phosphorylated on threonine and serine residues in a regulatory domain in the N-terminus [30], [31], and it must be correctly trafficked to the apical membrane of the DCT. In our study, we did not measure NCC phosphorylation or define localization to a specific sub-cellular compartment. Measurements of NCC phosphorylation are increasingly used as a surrogates for those of transport, yet evidence suggests that although phosphorylation is necessary, it is not sufficient, to explain transport activation in cation-chloride co-transporters [32]. Thus, we consider that the increase in total NCC expression, which paralleled the blood pressure rise, was more than sufficient to account for the increased thiazide-sensitive sodium reabsorption observed.

Previous studies have shown that both angiotensin II and aldosterone can independently increase NCC-mediated sodium transport in the DCT [12], [33], [34], [35], [36]. Our data show that the activation of NCC occurs via AT1R as losartan caused a natriuresis and fully normalized NCC activity. In contrast, spironolactone, at levels sufficient to achieve complete mineralocorticoid receptor blockade [37], had no effect on thiazide-sensitive sodium reabsorption or blood pressure. MR activation does not therefore appear to be major factor in our model, consistent with some previous studies [3], [6], [27], but contrasting with others [38]. It is also reported that hypertension *per se* reduces NCC expression in the apical membrane of the DCT [39]. We cannot discount a countervailing influence of hypertension on NCC activity, but our data indicate that the overall outcome was an angiotensin II-mediated increase in thiazide-sensitive sodium transport.

Chronic infusion of either hydrochlorothiazide or losartan was an effective antihypertensive measure. Both treatments also increased fractional sodium excretion, implying that renal sodium retention is a key hypertensive mechanism in this model. In support of this hypothesis, reciprocal transplantation studies in AT1AR null and wild-type mice show that renal AT1R mediate the chronic hypertensive effects of angiotensin II infusion by promoting renal sodium reabsorption [40]. However, in our study there was a trend towards volume contraction as blood pressure rose, suggesting that the effects of angiotensin II and dietary salt on blood pressure do not necessarily reflect volume expansion here [19], [20]. Indeed, other studies indicate that the antihypertensive effect of thiazide diuretics in angiotensin II-dependent hypertension is not exclusively related to depletion of plasma volume [19] and thiazides might have beneficial effects on vascular resistance [41].

### Renal microvascular injury

The afferent arteriole, glomerulus and post-glomerular structures were relatively protected from injury throughout the experiment: mild albuminuria developed by day 4 and was further increased coincident with raised renal vascular resistance. Our data suggest that protection from barotrauma reflects maintenance of efficient autoregulation [42]. In contrast, injury to the larger pre-glomerular resistance arteries was evident on the second day, before elevation of blood pressure into the hypertensive range. Renal injury was consistent with vasospasm, and might reflect damaging intermittent surges in systolic blood pressure [43]. These were not visualized in our telemetry analysis but data were only acquired hourly for short periods.

Pressure-independent renal injury, involving both angiotensin II and aldosterone [44], [45], [46] is also possible. The extent of pressure-independent renoprotection following RAAS inhibition is contentious [47] as AT1R blockade has exacerbated organ damage in some clinical trials [48]. Animal models suggest that transmission of high pressure to the kidney drives vascular remodeling and injury in angiotensin II-dependent hypertension [49], [50]. Our data show a protective effect of AT1R blockade, perhaps partially due to losartan's antihypertensive effect. However, chronic thiazide therapy, which caused a similar fall in blood pressure, did not abolish vascular injury, supporting a direct role for angiotensin II in end-organ injury. The beneficial effects of spironolactone were more clearly independent of blood pressure. Experimental and clinical data suggest that aldosterone, some of which may be synthesized in the kidney itself [51], is directly fibrogenic [52] and can damage renal structures [53]. Aldosterone-induced podocyte injury [54], for example, may explain the beneficial effect of spironolactone on the albuminuria seen here.

### Concluding Remarks

Renal, vascular and endocrine measurements have established distinctive aetiologies of hypertensive and renovascular disease following the conditional over-expression of renin in *cyp1a1-Ren2* TGR. Our data show that angiotensin II, aldosterone and sodium status act in concert to cause hypertension and renal impairment and underscore the key role of thiazide-sensitive sodium transport in the long-term regulation of blood pressure. Approximately 1/3^rd^ of the blood pressure rise was resistant to thiazide or losartan treatment. A similar proportion of hypertension persists in AT1R null mice infused with angiotensin II [46], pointing to non-renal, non-AT1R-mediated mechanisms. These may involve direct, detrimental actions of renin on vascular function [55], sensitization of the vasculature to vasoactive agents [23] and activation of the sympathetic nervous system [56].

## Materials and Methods

### Ethics Approval

Experiments were performed under a UK Home Office Project License and protocols were approved by The University of Edinburgh. All surgery was performed under anaesthesia (details are given under relevant sections, below) and all efforts were made to minimize suffering.

Experiments were performed on male *cyp1a1-Ren2 TGR* on a Fischer (F344) background, from a colony maintained in the university's animal house. Rats, aged 12–14 weeks, were given free access to water and commercial rat chow, containing 0.3% sodium by weight (Special Diet Services, UK) and were maintained under controlled conditions of temperature (21±1°C), humidity (50±10%) and light/dark (light 7am–7pm).

### Blood pressure measurement in conscious rats

Radiotelemetry devices (Model TA 11PAC20, Data Sciences, UK) were implanted into the thoracic aorta of *Cyp1a1-Ren2* TGR (n = 5) under ketamine/medetomidine anaesthetic, with buprenorphine used as a reversal agent and analgesic. After recovery, one-week equilibration was given during which restoration of the circadian rhythms for blood pressures, activity and heart rate was confirmed. Experimental data were then recorded and decoded using Art 4.0 software (Data Sciences, UK).

Baseline readings were taken over four consecutive days and, on day 5, the *cyp1a1-Ren2* transgene was induced by administering the naturally occurring xenobiotic, indole-3-carbinol (I3C; 100 mg/kg/day in vegetable oil), by gastric gavage at 10am; this was repeated on the following 6 days. Data were obtained on the hour, processed to means, smoothed using a 5-point rolling average and used to observe circadian patterns in blood pressure, activity and heart rate (for Figure 1). To analyze statistically periodicity, hourly means were smoothed by 3^rd^ order local polynomial regression, de-trended and analyzed using the χ^2^-periodogram algorithm [57].

### Renal function in anaesthetized rats

Separate cohorts of *cyp1a1-Ren2* TGRs received I3C for either 1, 3 or 7 days; the control group (n = 8) received an equivalent volume of vegetable oil over a 7-day period. Time-points are designated as control (n = 8), day 2 (n = 9); day 4 ( = 9) and day 8 (n = 9). In each group, mean arterial blood pressure and renal function were measured twenty-four hours after the final gavage treatment, as follows. Following thiobutabarbital anaesthesia (Inactin, 120 mg/kg IP; Sigma Aldrich UK), a cannula was inserted into the right jugular vein through which a solution, containing (in mmol/l): 120 NaCl, 10 LiCl, 15 NaHCO_3_ and 5 KCl, was infused (1 ml/h/100 g IV). The infusate also contained 0.5% p-amino hippuric acid (PAH) and 0.5% FITC-inulin. A cannula, containing heparin-saline, was placed in the right carotid artery for collection of blood and measurement of mean arterial blood pressure. The bladder was catheterized for collection of urine and a tracheostomy was performed to maintain a clear airway.

One hour after the completion of surgery, rats underwent the following protocol: three consecutive collections of urine were made, each of 60 minutes. A sample (75 µl) of arterial blood was drawn at the start and then after each urine collection period for the measurement of haematocrit, FITC-inulin and PAH.

After the first urine collection, amiloride was administered (2 mg/kg bolus; 2 mg/kg/h infusion). After the second urine collection, hydrochlorothiazide was administered (2 mg/kg bolus; 2 mg/kg/h infusion) alongside the amiloride. The drugs were delivered in a DMSO vehicle (2% v∶v), which, in a separate group of rats (n = 7), had no effect on renal function or blood pressure (data not shown). Finally, a 1 ml sample of arterial blood was taken for measurement of plasma electrolytes and the kidneys frozen at −80°C and used for Western blot. Animals were then killed by an overdose of anaesthetic.

### Analysis & calculations

The concentration of Na, K and Li in plasma and urine was measured using ion-selective electrodes (Roche ISE, model 9180). The effect of amiloride on sodium excretion was taken as the difference between excretion rates in the first and second urine collections; that of hydrochlorothiazide was taken as the difference between excretion rates in the second and third urine collections.

FITC-inulin and PAH concentrations were measured as described [4]. The clearance of PAH was taken as effective renal plasma flow and used to calculate renal blood flow. Renal vascular resistance was taken as the quotient of arterial blood pressure and renal blood flow. Lithium clearance was used as an index of fluid delivery to the end of the proximal tubule [26].

### Urine collection in conscious rats

*Cyp1a1-Ren2* transgenic rats (n = 8) were housed individually in metabolism cages with free access to powdered rat chow and water. Rats were acclimatized and after a 4-day control period, I3C was administered on each of 7 consecutive days, as described above. Urine was collected every 24-hours. Albumin was measured using commercial assays (Alpha Laboratories Ltd., UK), aldosterone by ELISA [58].

### Interventional studies

Hydrochlorothiazide (4 mg/kg/d; n = 6), spironolactone (20 mg/kg/d; n = 6) or losartan (10 mg/kg/d, n = 6) were administered by osmotic minipump (Alzet, Model 2ML1, Charles River UK), implanted on day 0 under isofluorane anaesthesia. A control group of rats (n = 6) received vehicle (50% DMSO, 50% saline) alone. After implant, the *Ren2* transgene was induced over a period of three days before being anesthetized for renal function studies, as described. A three-day induction was selected as a time-point at which significant changes to tubular function were not associated with a decline in renal haemodynamics or major destructive vascular lesions.

### Western blot analysis

Total protein was extracted from one kidney by homogenization and differential centrifugation in ice-cold buffer, containing 250 mM sucrose, 10 mM triethanolamine and 2% protease inhibitor cocktail, pH 7.6 (Pierce Protein Research, Thermoscientific, UK). Protein solubilized in sample buffer (50 µg of total protein per lane) was separated by SDS-PAGE and blotted to PVDF membrane by semi-dry transfer. Immunoblotting was performed using polyclonal antibodies against NCC (Chemicon, UK; 1∶2500) and αENaC (Upstate, NY, USA; 1∶2000) and horseradish peroxide-conjugated secondary antibodies. Immunodetection was quantified by densitometry using ImageJ after treatment of the blots with ECL reagents. Equal loading of total protein was confirmed in the GAPDH (R&D Systems, UK; 1∶5000) immunoblot.

### Histopathologic analysis

Kidneys were immersion-fixed in 10% neutral buffered formalin for 48 h followed by paraffin embedding. Four-micron sections were examined blind by a Consultant Pathologist (C.O.B.). Kidneys from at least three rats per experimental group were examined. For each, 2 complete hemisections of the left kidney were blocked and all vascular profiles in each hemisection examined at 4 different levels in the block. Two were stained with hematoxylin and eosin and two with Periodic Acid Schiff, giving 8 levels per case. Analysis of microvascular injury was performed using an ordered categorical scale according the presence of destructive microvascular arterial or arteriolar lesions characterized by intramural necrosis and/or fibrinoid change. A score of “one” indicates non-confluent necrosis and isolated myocyte death; “two” indicates a single foci of necrosis per section; “three” indicates 2–3 foci; and “four” indicates >3 foci of destructive vascular lesions. Undamaged sections were scored zero.

### Statistics

Data are presented as mean ± SE. Comparisons were made using either one- or two-way analysis of variance, as appropriate: post-hoc comparisons were made using the Holm-Sidak test. For scoring of microvascular injury, contingency tables were generated for X^2^ analysis.
